# Automatic detection and counting of urediniospores of *Puccinia striiformis* f. sp. *tritici* using spore traps and image processing

**DOI:** 10.1038/s41598-018-31899-0

**Published:** 2018-09-11

**Authors:** Yu Lei, Zhifeng Yao, Dongjian He

**Affiliations:** 1 College of Mechanical and Electronic Engineering, Northwest A&F University, Yangling, Shaanxi 712100 China; 2 Key Laboratory of Agricultural Internet of Things, Ministry of Agriculture and Rural Affairs, Yangling, Shaanxi 712100 China; 3 Shaanxi Key Laboratory of Agricultural Information Perception and Intelligence Service, Yangling, Shaanxi 712100 China

## Abstract

The quantitative monitoring of airborne urediniospores of *Puccinia striiformis* f. sp. *tritici* (*Pst*) using spore trap devices in wheat fields is an important process for devising strategies early and effectively controlling wheat stripe rust. The traditional microscopic spore counting method mainly relies on naked-eye observation. Because of the great number of trapped spores, this method is labour intensive and time-consuming and has low counting efficiency, sometimes leading to huge errors; thus, an alternative method is required. In this paper, a new algorithm was proposed for the automatic detection and counting of urediniospores of *Pst*, based on digital image processing. First, images of urediniospores were collected using portable volumetric spore traps in an indoor simulation. Then, the urediniospores were automatically detected and counted using a series of image processing approaches, including image segmentation using the *K*-means clustering algorithm, image pre-processing, the identification of touching urediniospores based on their shape factor and area, and touching urediniospore contour segmentation based on concavity and contour segment merging. This automatic counting algorithm was compared with the watershed transformation algorithm. The results show that the proposed algorithm is efficient and accurate for the automatic detection and counting of trapped urediniospores. It can provide technical support for the development of online airborne urediniospore monitoring equipment.

## Introduction

Wheat stripe (yellow) rust, which is caused by *Puccinia striiformis* f. sp. *tritici* (*Pst*), is a wheat disease that is prevalent across the world, particularly in cool and moist regions^1,2^. It is one of the most important and devastating airborne wheat diseases in China and has caused severe yield reduction, resulting in significant economic loss^3^. Urediniospores of *Pst* are heteroecious macro-cyclic rust pathogens that require a living host (wheat/grasses, Berberis/Mahonia spp.) to complete the asexual and sexual phases of their life cycle^4–6^. Urediniospores maintain the dominant asexual stage of the pathogen population on the primary hosts. This is the main period for the wide-scale stripe rust epidemics reported on wheat^2^. Urediniospores have the capacity for long-distance dispersal by wind movement, which may extend to hundreds and perhaps thousands of kilometres from the centre of origin of *Pst*^7–9^. Urediniospores of *Pst* infect wheat as a result of urediniospore deposition by dispersed air or raindrops on the leaf surface^10^. Therefore, the aerial dispersal of *Pst* urediniospores is the main reason for the occurrence and prevalence of the disease of wheat stripe rust. The occurrence of the disease is closely related to the number of urediniospores in wheat field air. The efficient capturing and quantitative monitoring of urediniospores in the atmosphere of a wheat field can provide critical information for airborne wheat disease prediction.

Because of the improvement and popularization of spore trap devices, airborne fungal spores are commonly collected by these devices^11–13^. Previous studies have often used them for sampling onto microscope slides or plastic tape, which were coated with a thin film of petroleum jelly and then examined microscopically at hundredfold magnification. The traditional microscopic spore counting method mainly relies on naked-eye observation by professional and technical personnel in the laboratory. Because of the great number of trapped spores, this method is labour-intensive and time-consuming and has low efficiency, sometimes leading to huge errors. Thus, the timely identification and counting of trapped spores is hindered. Studies on the application of molecular-biological techniques to detect and quantify fungus have been reported^14–16^. However, it is difficult to translate the techniques to practical applications because of the high technical requirements and great operational complexes.

Machine vision techniques have been widely used in the automatic diagnosis and grading of plant diseases in recent years. Several algorithms for the digital image process have been used in studies on plant diseases. Image recognition of plant diseases^17–19^ and the automatic classification of disease severity^20–22^ can be achieved using appropriate image processing technologies. Recently, some studies on the automatic counting of fungal spores with using micro-image processing have been reported. Li *et al*.^23^ proposed a method based on the watershed transformation algorithm to count the urediniospores of *Pst*. However, the results appeared to incorrectly split the positions of the spores and demonstrated over-segmentation when the urediniospores had rough boundaries or a complicated touching condition. Chesmore *et al*.^24^ developed an image analysis system for rapidly discriminating between *T*. *walkerii* and *T*. *indica*. Principal components analysis was performed on many parameters, including the perimeter, surface area, number of spines, spine size, maximum and minimum ray radii, aspect ratio and roundness, to obtain a linear separation of species. The analysis system achieved 97% accuracy for separating *T*. *walkerii* and *T*. *indica*. Wang *et al*.^25^ proposed a new method based on image processing and artificial neural network to automatically detect powdery mildew spores. The 63.6% correct rate of testing pictures showed that it could achieve the automatic detection and counting of powdery mildew spores. Xu *et al*.^26^ used a computer-assisted digital image processing method, which obtained the spore image exterior outline characteristics for spore type analysis and automatic counting. All previous works demonstrated excellent performance. However, when the spores largely touched each other or there was no strong gradient existing in the touching spores, these studies usually had the problem of over-segmentation or under-segmentation. In reality, in-field trapped pathogen spores may touch or even overlap. Under the touching condition, it is difficult to separate the touching spores, which may affect the counting accuracy. Therefore, the touching spores in the image need to be split into separate spores. A variety of segmentation algorithms for touching objects in images, such as cells^27–29^, fruits^30,31^, and cereals^32–34^, have been reported, and there have been some achievements in these fields. However, these algorithms are not suitable for the segmentation and counting of spores. Hence, it is necessary and urgent to develop a new method for the rapid and accurate identification and quantification of spores.

On the basis of simulating the wheat field environment, this study develops a new algorithm for the automatic detection and counting of trapped pathogen urediniospores of *Pst*. First, the urediniospores are segmented using the *K*-means clustering algorithm and converted into a binary image. Second, to remove noise, pre-processing measures, including region filling, small area removal and open operations, are implemented to fill the spores, filter noise and smooth the urediniospore edges. Third, a feature combination of shape factor of target contour and area is selected as the basis for the discrimination of touching urediniospores. Fourth, the contour of touching urediniospores is extracted and divided into several contour segments based on concavity. Then, the contour segments that belong to the same urediniospores are merged, which is the main issue to solve. In this section, the distance measurement is proposed to eliminate the segments that have a low possibility of belonging to the same urediniospore. The candidate ellipse is then fitted using the least-squares ellipse fitting algorithm, and the deviation error measurement is used to determine its fitness evaluation. If the contour segments satisfy the conditions of the distance and the deviation error measurement, they are merged to create a new segment. Finally, the ellipses are fitted with these new segments as the best representative ellipses for the touching urediniospores. The objective of this study is to develop a new algorithm for the automatic detection and counting of trapped pathogen spores, and to assess the algorithm’s detection accuracy by comparing the automatic counting algorithm with the watershed transformation algorithm.

## Results

To verify the validity and accuracy of the proposed automatic counting algorithm in this paper, the counting test was performed on a total of 120 urediniospore images. To accurately obtain the real number of urediniospores, the results of a manual counting method were used as the actual number of urediniospores. The performance of the proposed algorithm was compared with that of the watershed transformation algorithm^23^. A personal computer with a 3.3 GHz processor and 8 GB RAM was used as the hardware part of the computer vision system, and all the algorithms were developed in MATLAB R2014a (The MathWorks Inc, Natick, MA, USA). The examples are demonstrated below.

**Figure 1:**
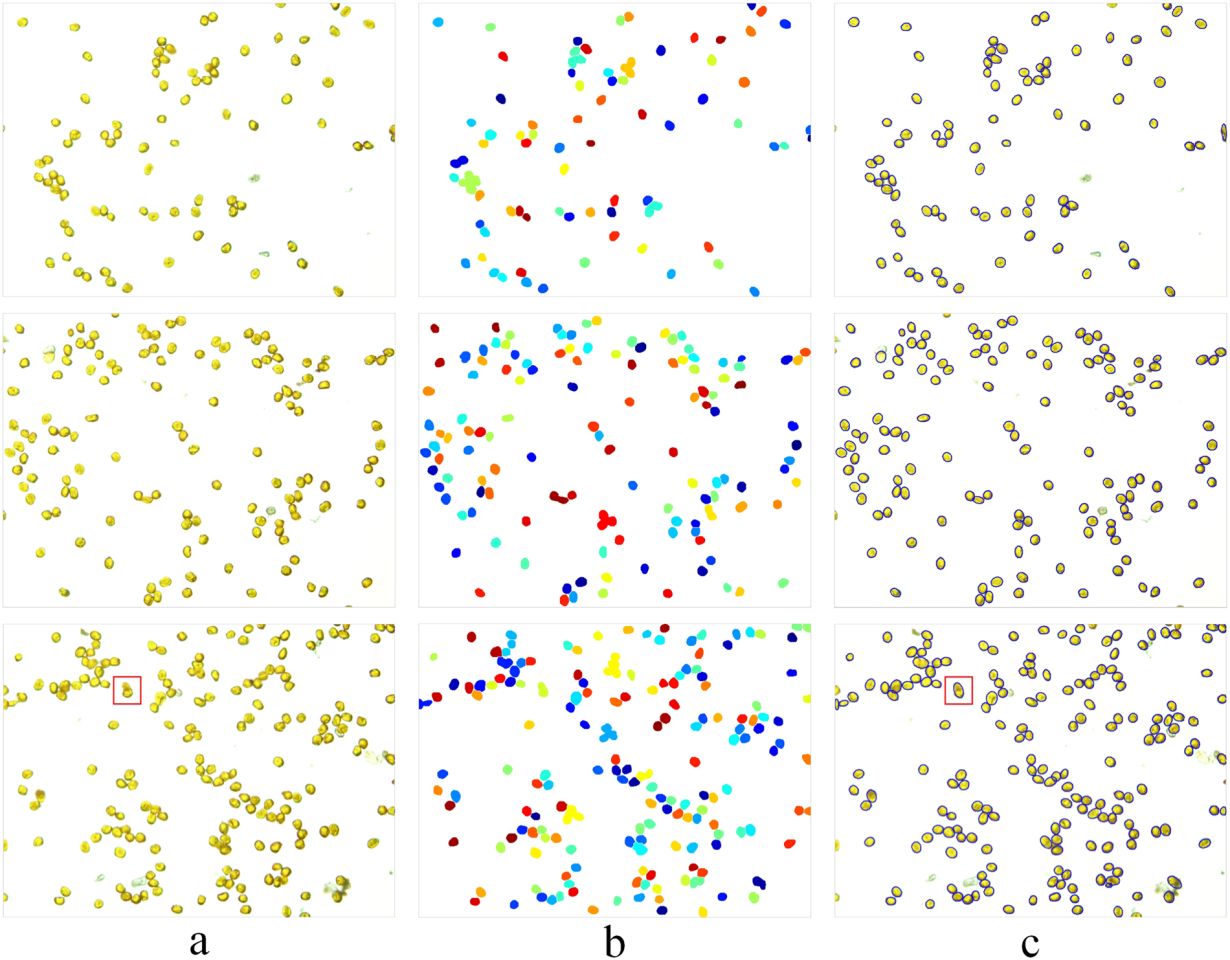
Segmentation and counting results of the part of test images: (**a**) original digital image of urediospores; (**b**) segmentation effect and counting results based on the watershed segmentation algorithm; (**c**) segmentation effect and counting results based on the proposed algorithm.

A comparison of the results processed by the proposed algorithm and the watershed transformation algorithm is shown in Fig. 1. As shown in Fig. 1a, there were not only individual urediniospores but also touching clumps and even some occluded urediniospores, which may not have been possible to separate correctly using previous techniques. Figure 1b shows the result processed by the watershed transformation algorithm, in which the cases of two urediniospore adhesions and several urediniospores connected in series were well separated. However, it appeared to be over-segmented when the urediniospores had rough boundaries or under complicated touching/cluster conditions, and some of the segmentation locations were not accurate, which made the counting accuracy low. Figure 1c shows the result processed by the proposed algorithm, in which the cases above were well separated. Moreover, the proposed algorithm was robust, because it performed well, even when the contours of touching urediniospores were irregular.

To evaluate both methods, the counting accuracy of detection was used, which was the ratio of the number of correctly detected urediniospores to the total number of urediniospores in the input image. Comparisons of the counting accuracy results are shown in Table 1. The lowest counting accuracy of the proposed method was 92.7%. The highest counting accuracy was 100%. The total average counting accuracy was 98.6%, which was an increase of 6 percentage points over that of the watershed transformation algorithm (92.6%). The experimental results of the lowest accuracy, highest accuracy and total average accuracy show that the proposed algorithm had better validity and correctness than the watershed transformation algorithm for urediniospore image segmentation and counting. Therefore, the proposed method is efficient and accurate for the automatic detection and counting of trapped urediniospores.

**Table 1:** Comparisons of counting accuracy of two different segmentation algorithms.

Range of spores in one image	Number of samples	Watershed transformation algorithm			Proposed algorithm		
		Lowest accuracy/%	Highest accuracy/%	Average accuracy/%	Lowest accuracy/%	Highest accuracy/%	Average accuracy/%
9 ~ 59	48	81.3	106	95.3	95.5	100	99.5
60 ~ 110	37	85.9	101	94.7	94.9	100	98.9
111 ~ 161	22	84.2	100	92.1	93.2	100	98.2
162 ~ 177	13	82.7	97.2	88.4	92.7	100	97.7
Total	120	83.5	101	92.6	93.6	100	98.6

## Discussion

**Figure 2:**
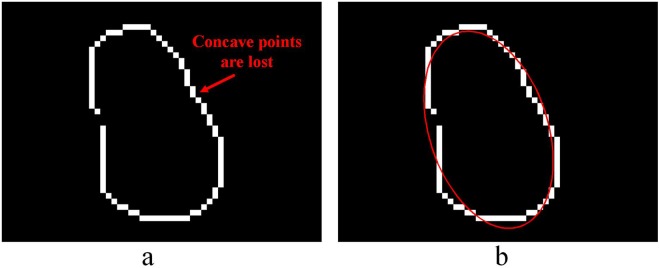
Example of overlapping urediniospore segmentation: (**a**) contour segments; **(b**) separation result.

It is inevitable that overlap will arise while capturing the urediniospores of wheat stripe rust. As shown in the red boxes in Fig. 1a and c, the urediniospore in the upper part of the occluded urediniospores is blurred, because of the shallow depth of field under high optical magnification. In this case, the concave point may be lost at the contour of the concave object (Fig. 2a), which leads to a segmentation fault and affects the accuracy of automatic counting. From Fig. 2b, two urediniospores are misdivided into one urediniospore by the ellipse fitting algorithm. Thus, further research is needed to solve the problem of the segmentation count for overlapping urediniospores.

Currently, we mainly focus on the automatic detection and counting of pathogenic urediniospores of Pst that are trapped using a spore trap device via an indoor simulation. The scenarios are more complicated in a wheat field environment. Some atmospheric particles, such as pollen and other fungal spores, may also affect the accuracy of the automatic counting of urediniospores. Thus, further work is required to improve the counting accuracy of urediniospores that are collected in an actual farmland environment.

## Materials and Methods

### Materials

Fresh urediniospores of Pst were selected as the experimental objects and obtained from the College of Plant Protection at Northwest A&F University, Yangling, Shaanxi, China. The other materials and instruments included petroleum jelly, microscope slides, an aurilave, portable volumetric spore traps (TPBZ3, Zhejiang Top Instrument Co., Ltd, China), microscope (BX52, Olympus, Japan) and a desktop computer.

### Image acquisition

**Figure 3:**
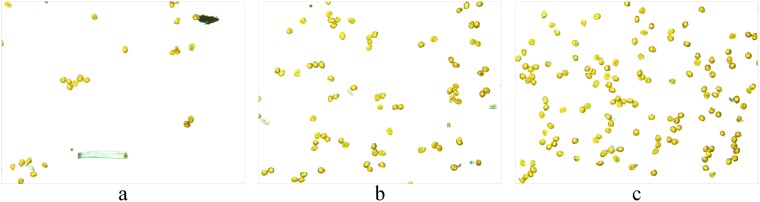
Original RGB images for different capture times: (**a**) image after capturing for 5 min; (**b**) image after capturing for 10 min; (**c**) image after capturing for 15 min.

The experiment was conducted in the laboratory of the College of Mechanical and Electronic Engineering at Northwest A&F University, Yangling, Shaanxi, China. The collection of airborne fungal urediniospores was conducted by simulating the wheat field environment. Portable volumetric spore traps were used to collect airborne urediniospores of Pst. In the laboratory, an aurilave was used first to slowly and continuously blow the urediniospores above the trap to spread them into the air. Airborne particles were then deposited onto microscope slides that were uniformly coated with a thin film of petroleum jelly. To obtain different densities of urediniospores on the slides, urediniospores were collected continuously for 5, 10 or 15 mins and repeated 10 times to obtain 30 slides containing different urediniospore densities. The fungal urediniospores on the slides were observed and photographed at the genus level with the aid of a light microscope under ×200 magnification. All the 150 experimental images (4,140 × 3,096 pixels) of urediniospores (five points each slide under the microscope were randomly selected to take images) were acquired for the validation of the proposed algorithm using a digital imaging system (DP72, Olympus, Japan) of 72 dpi at 24 bits using the RGB model. Thirty images were randomly selected for algorithm training, and the remaining 120 images were used for testing. Partial original images are shown in Fig. 3. The urediniospores were broadly ellipsoidal to broadly obovoid, with a mean of 24.5 × 21.6 μm, and yellow to orange. A large number of urediniospores in the images touched each other, which made it difficult to automatically count them.

### Segmentation of the urediniospore images using the *K*-means clustering algorithm

Image segmentation from the background is an important step in the image processing technique. Urediniospore target segmentation can be viewed as a clustering problem in the case in which the classification of the pixel points is unknown and the image can be divided into several regions based on the characteristic value of the pixel points. The *K*-means clustering algorithm is an unsupervised partitioning algorithm that divides data into a predetermined class *k* to minimise the error function and is widely used in image segmentation fields^35,36^. It is accurate and highly efficient for largescale data processing, which makes it more suitable to segmenting urediniospore images in this work. Because only the urediniospore region was required in this research, *k* = 2 was used, and the original image was divided into two classes: the urediniospore region and the background region.

**Figure 4:**
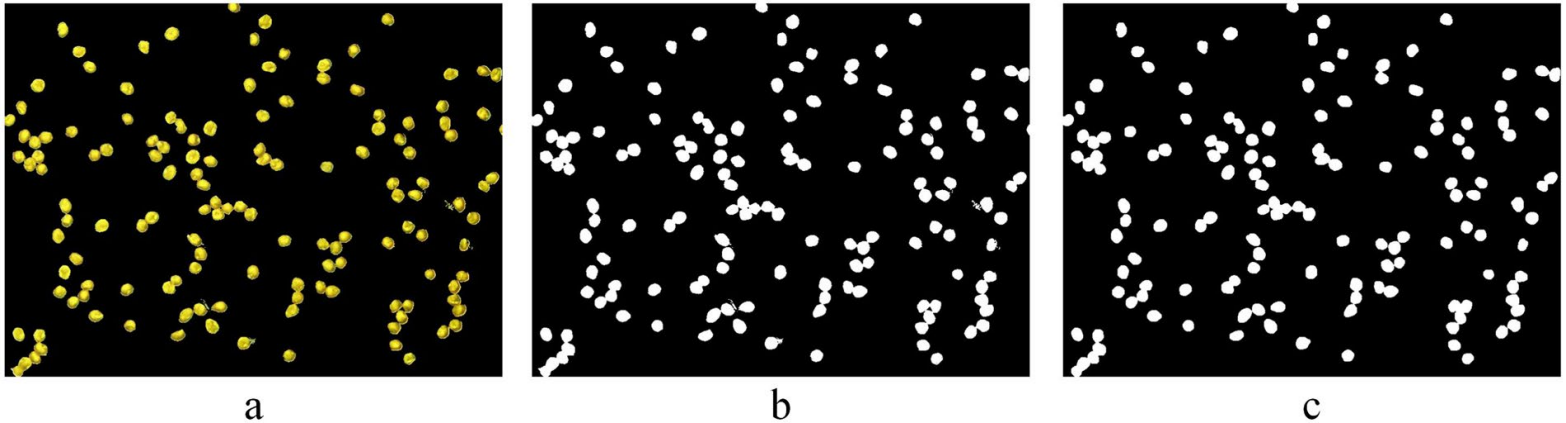
Results of the urediniospore image after the *K*means algorithm and preprocessing: (**a**) extracted image of urediniospores after the *K*-means algorithm with *k* = 2; (**b**) binary image of the extracted urediniospores using the fixed threshold algorithm; (**c**) binary image after morphology preprocessing.

The size of images we acquired was large, and it was time consuming to process and segment them. First, we resized them to 911 × 682 pixels. The colour space was transformed from the RGB colour space to the L*a*b* colour space. The *K*means clustering algorithm was then used to cluster the urediniospore images into two classifications based on the a* and b* colour components. Figure 3c is the original image. Figure 4a shows the processing results of *K*-means clustering, demonstrating that it could be applied for image segmentation. Figure 4b is the binary image of Fig. 4a using the fixed-threshold algorithm based on the threshold of 0.2. From Fig. 4b, we can observe that it contained noise, such as holes and spurs, which affected the accuracy of the subsequent contour detection and urediniospore count. Therefore, further processing was needed.

### Pre-processing of the urediniospore image

To remove noise, pre-processing measures, including region filling, small-area removal and open operations, were implemented to fill the spores, filter noise and smooth the spore edges. First, the holes in the urediniospores were filled using the operation of region filling. Second, the areas of less than 190 pixels, that is, small particles, were removed using the area opening operation. At the moment when the areas of the urediniospores on the boundary of the image were less than 190 pixels, the urediniospores were removed from the binary image and counted as the urediniospores of the other images. Finally, the morphological opening operation with a ‘disk’-shaped structural element with a radius of 5 pixels was performed to remove spurs and smooth the urediniospore contour. As a result, the processed image (Fig. 4c) showed no noise and the contour became smoother, which was helpful in subsequent processing.

### The identification of touching urediniospores based on the shape factor and area

Figure 4c shows that the binary image included not only individual urediniospores but also two or more touching urediniospores. The regional contour of the touching urediniospores was more complex and larger than that of the individual urediniospores. Thus, a feature combination of the shape factor of the target contour and area was selected as the basis for the discrimination of touching urediniospores. The shape factor (*SF*) formulas^37^ can be defined as

1 $$SF=4\pi S/{L}^{2},$$

where *S* is the area pixel value of a connected region and *L* is the perimeter pixel value of a connected region.

**Figure 5:**
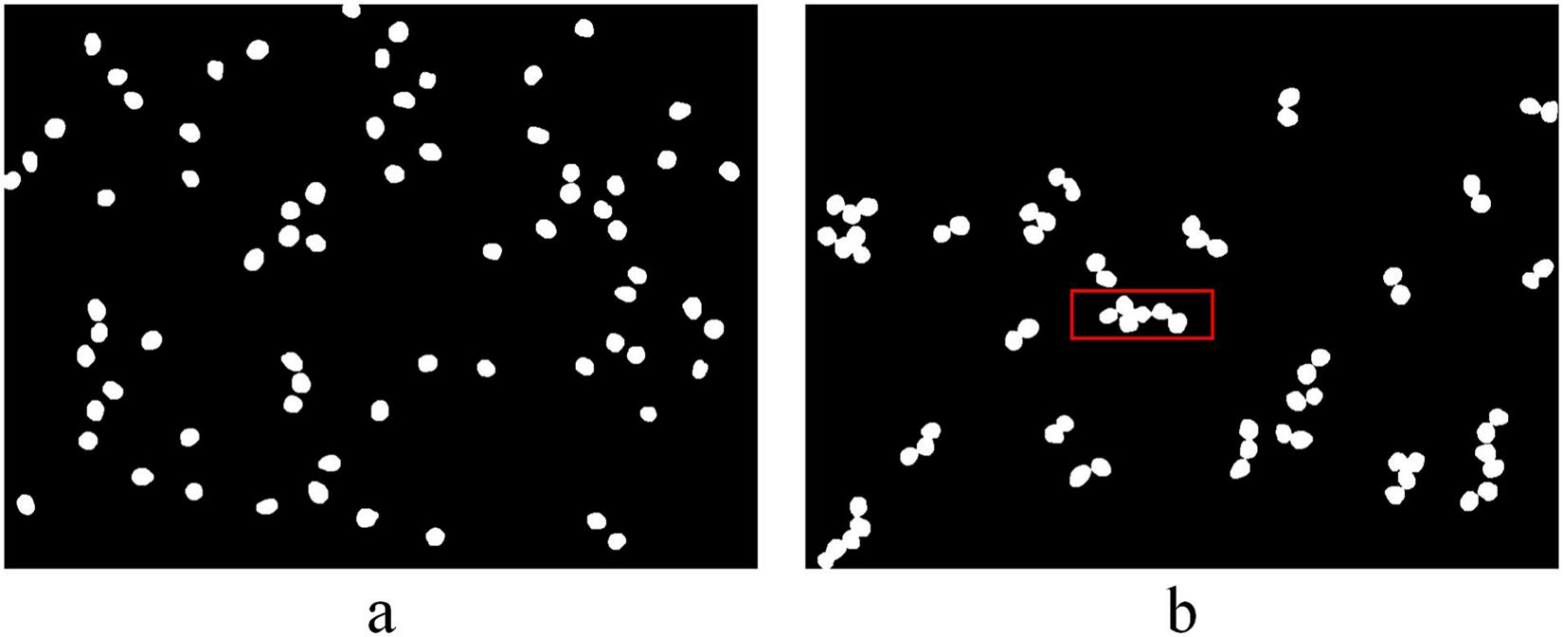
Binary images of individual and touching urediniospores: (**a**) binary image of individual urediniospores; (**b**) binary image of touching urediniospores.

The contour of the touching urediniospores was larger and more complicated than that of the individual urediniospores after sampling statistics because it was concave, and the shape factor was smaller. When 0.9080 < *SF* < 1.0912 and 200 < *S* < 523, the objects were identified as individual urediniospores. When 0.2625 < *SF* < 0.7606 and 600 < *S* < 2301, the objects were identified as touching urediniospores. Thus, *SF* and *S* were set to be 0.8 and 560 according to various tests. Let *i* be a connected region. If

2 $$S{F}_{i} > S{F}_{0}\,\& \,{S}_{i} < {S}_{{\rm{\max }}}.$$

is satisfied, region *i* is assessed to be an individual urediniospore, otherwise it is assessed to be touching urediniospores. Figure 5 shows the result of touching urediniospore identification for Fig. 4c, where the individual urediniospores and touching urediniospores were correctly discriminated.

### Touching urediniospore contour segmentation based on concavity

From the above processing step, if the region was identified as an individual urediniospore in the binary image, then the ellipse number was directly counted by the least-squares ellipse fitting method. To automatically count the spores precisely, these touching urediniospores must be separated, which is the main contribution of this work.

**Figure 6:**
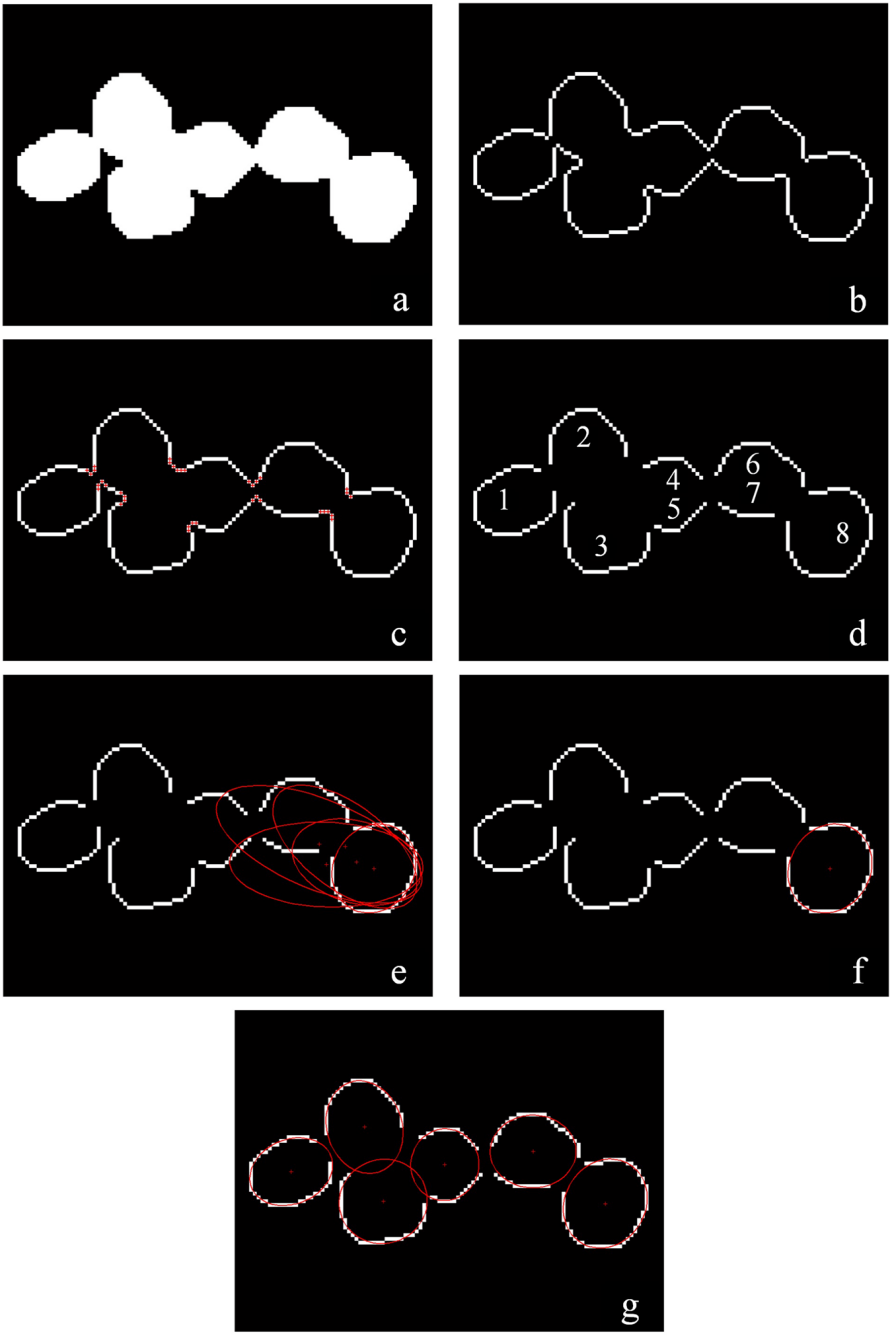
Processing example of the proposed algorithm: (**a**) binary image after pre-processing; (**b**) contour of touching urediniospores; (**c**) red concave points on the contour; (**d**) contour segments; (**e**) candidate ellipses; (**f**) best representative ellipse of one urediniospores; (**g**) final separation result.

As an example, the contour of the touching urediniospores in the red rectangular box in Fig. 5b was extracted by the canny edge detector, which is shown after enlargement in Fig. 6b, and the contour points were stored in an ordered list. The following detection method for the concavity was used.

Let *p*_*t*_(*x*_*t*_, *y*_*t*_) be a contour point, and the angle between the vectors ***p***_*t*_***p***_*t−h*_ and ***p***_*t*_***p***_*t*+*h*_ be defined as the concavity of *p*_*t*_, where *p*_*t−h*_ and ***p***_*t*+*h*_ denote the *h*th adjacent contour points of ***p***_*t*_; *h* was set to 3 according to the pretest. The formula for concavity can be expressed as

3 $${\rm{concavity}}\,({p}_{t})={\rm{angle}}\,({p}_{t-h}{\boldsymbol{,}}\,{p}_{t}{\boldsymbol{,}}\,{p}_{t+h})=\arccos \frac{{{\boldsymbol{p}}}_{t}{{\boldsymbol{p}}}_{t-h}\cdot {{\boldsymbol{p}}}_{t}{{\boldsymbol{p}}}_{t+h}}{||{{\boldsymbol{p}}}_{t}{{\boldsymbol{p}}}_{t-h}||\cdot ||{{\boldsymbol{p}}}_{t}{{\boldsymbol{p}}}_{t+h}||}$$

If the point is determined to be the concave point, then it needs to meet the following two conditions (Fig. 6c): concavity(*p*_*t*_) is in the range angle(*δ*_1_,*δ*_2_); line $\overline{{p}_{t-h}{p}_{t+h}}$ does not traverse the touching urediniospores.

With 30 urediniospore image samples for statistical analyses, the angles of the concave point *p*_*t*_ between the vectors ***p***_*t*_***p***_*t*−*h*_ and ***p***_*t*_***p***_*t* + *h*_ were in the range of 50° to 150°. Thus, the values of *δ*_1_ and *δ*_2_ were set to 50° and 150°, respectively.

With these concave points, the contour (*C*) of the touching urediniospores was divided into *N* contour segments and *K* concave points (Fig. 4d):

4 $${C}={C}{{S}}_{1}+\ldots +{C}{{S}}_{i}+\ldots +{C}{{S}}_{N}+{c}{{p}}_{1}+\ldots +{c}{{p}}_{j}+\ldots +{c}{{p}}_{K}$$

The contours of the touching urediniospores were stored in an ordered cell structure using the 8-connected boundary tracking method and then used for subsequent urediniospore contour segment merging.

### Touching urediniospore contour segment merging

After segmentation in the above processing step, one contour of the same urediniospores may be divided into several contour segments. The aim of this subsection is to merge the contour segments that belong to the same urediniospores. First, several measurements and the ellipse fitting algorithm are proposed, and then the merging steps are explained.

#### Distance measurement between contour segments

If the distance between two contour segments is large, then the possibility that these two segments belong to the same urediniospore is low and vice versa. Thus, according to this theory, a method based on the distance measurement is used to express the relevance between these contour segments to eliminate the segments that had a low probability of belonging to the same urediniospore. The distance between two contour segments can be described by as

5 $$DM({C}{{S}}_{{i}}{\boldsymbol{,}}{C}{{S}}_{{j}})=\frac{[d({p}_{i1}{\boldsymbol{,}}{p}_{j1})+d({p}_{i1}{\boldsymbol{,}}{p}_{j{M}_{j}})+d({p}_{i{m}_{i}}{\boldsymbol{,}}{p}_{j{m}_{j}})+d({p}_{i{M}_{i}}{\boldsymbol{,}}{p}_{j1})+d({p}_{i{M}_{i}}{\boldsymbol{,}}{p}_{j{M}_{j}})]}{5},$$

where *d*(*p*_*i*1_,*p*_*j*1_), $d({p}_{i1}{\boldsymbol{,}}{p}_{j{M}_{j}})$, $d({p}_{i{M}_{i}}{\boldsymbol{,}}{p}_{j1})$, and $d({p}_{i{M}_{i}}{\boldsymbol{,}}{p}_{j{M}_{j}})$ are the Euclidian distances between the end points of contours *CS*_*i*_ and *CS*_*j*_, and $d({p}_{i{m}_{i}}{\boldsymbol{,}}{p}_{j{m}_{j}})$ is the Euclidian distance between the middle points of contours *CS*_*i*_ and *CS*_*j*_.

#### Ellipse fitting

Because the urediniospores in the image resembled an ellipsoidal shape, to separate the touching urediniospores, the least-squares ellipse fitting algorithm proposed by Fitzgibbon *et al*.^38^ was implemented to fit the contour of the urediniospores. A general ellipse can be described by an implicit second-order polynomial:

6 $$F({\boldsymbol{\alpha }},{\boldsymbol{X}})={\boldsymbol{X}}\cdot {\boldsymbol{\alpha }}=a{x}^{2}+bxy+c{y}^{2}+dx+ey+f=0,$$

with an ellipse constraint:

7 $$4ac-{b}^{2}=1,$$

where $${\boldsymbol{X}}=[\begin{array}{ccccc}{x}^{2} & xy & {y}^{2} & x & \begin{array}{cc}y & 1\end{array}\end{array}]$$ and $${\boldsymbol{\alpha }}={[\begin{array}{ccccc}a & b & c & d & \begin{array}{cc}e & f\end{array}\end{array}]}^{T}$$ are the coefficients of the ellipse, and *x* and *y* are coordinates of sample points lying on it.

Equation (7) can be represented in vector form:

8 $${{\boldsymbol{\alpha }}}^{T}C{\boldsymbol{\alpha }}=1,$$

where the constraint matrix ***C*** is of size 6 × 6, and

9 $${\boldsymbol{C}}{\boldsymbol{=}}[\begin{array}{cccccc}0 & 0 & 2 & 0 & 0 & 0\\ 0 & -1 & 0 & 0 & 0 & 0\\ 2 & 0 & 0 & 0 & 0 & 0\\ 0 & 0 & 0 & 0 & 0 & 0\\ 0 & 0 & 0 & 0 & 0 & 0\\ 0 & 0 & 0 & 0 & 0 & 0\end{array}].$$

Polynomial *F*(***α***, ***X***_*i*_) is called the algebraic distance of data point (*x*_*i*_, *y*_*i*_) to ellipse *F*(*x*_*i*_, *y*_*i*_) = 0. Because of sample point errors, the deviation resulted in *F*(*x*_*i*_, *y*_*i*_) ≠ 0. The fitting problem of the ellipse can be resolved by minimising the sum of the squared algebraic distances of the set of *N* data points to the ellipse:

10 $$E={\sum _{i=1}^{m}F({x}_{i}{\boldsymbol{,}}{y}_{i})}^{2},$$

which can be reformulated in vector form:

11 $$E={\Vert D{\boldsymbol{\alpha }}\Vert }^{2},$$

where the design matrix ***D*** of size N × 6 is

12 $${\boldsymbol{D}}=[\begin{array}{cccccc}{x}_{1}^{2} & {x}_{1}{y}_{1} & {y}_{1}^{2} & {x}_{1} & {y}_{1} & 1\\ \vdots  & \vdots  & \vdots  & \vdots  & \vdots  & \vdots \\ {x}_{i}^{2} & {x}_{i}{y}_{i} & {y}_{i}^{2} & {x}_{i} & {y}_{i} & 1\\ \vdots  & \vdots  & \vdots  & \vdots  & \vdots  & \vdots \\ {x}_{m}^{2} & {x}_{m}{y}_{m} & {y}_{m}^{2} & {x}_{m} & {y}_{m} & 1\end{array}].$$

Vector ***α*** can be calculated using the Lagrange coefficient and differential based on Equations (8) and (11), which leads to the following:

13 $$\{\begin{array}{c}W{\boldsymbol{\alpha }}=\lambda {\boldsymbol{C}}{\boldsymbol{\alpha }}\\ {{\boldsymbol{\alpha }}}^{T}{\boldsymbol{C}}{\boldsymbol{\alpha }}=1\end{array},$$

where ***W*** is the scatter matrix of size 6 × 6, and *λ* is an eigenvalue for ***W***:

14 $${\boldsymbol{W}}={{\boldsymbol{D}}}^{T}{\boldsymbol{D}}.$$

Equation (13) is a generalised eigenvector system. According to the generalised eigenvalue, the solution method can derive the following:

15 $${{\boldsymbol{\alpha }}}_{i}=(\sqrt{\frac{1}{{{\boldsymbol{u}}}_{i}^{T}{\boldsymbol{C}}{{\boldsymbol{u}}}_{i}}})\,{{\boldsymbol{u}}}_{i}=(\sqrt{\frac{{\lambda }_{i}}{{{\boldsymbol{u}}}_{i}^{T}{\boldsymbol{S}}{{\boldsymbol{u}}}_{i}}}){{\boldsymbol{u}}}_{i},$$

where *λ*_*i*_ and ***u***_*i*_ are the eigenvalue and eigenvector for Equation (13).

The sum of the squared algebraic distances of the points to the ellipse can be derived as

16 $$E={\Vert {\boldsymbol{D}}{\boldsymbol{\alpha }}\Vert }^{2}={{\boldsymbol{\alpha }}}^{T}{{\boldsymbol{D}}}^{T}{\boldsymbol{D}}{\boldsymbol{\alpha }}={{\boldsymbol{\alpha }}}^{T}{\boldsymbol{W}}{\boldsymbol{\alpha }}=\lambda {{\boldsymbol{\alpha }}}^{T}{\boldsymbol{C}}{\boldsymbol{\alpha }}=\lambda .$$

Thus, the chosen eigenvector ***α***_*i*_ (*i* is the eigenvector number) that corresponds to the minimal positive eigenvalue *λ*_*i*_ represents the best-fit ellipse for the given set of points.

#### Deviation error measurement from the contour segments to the fitted ellipse

When elliptical fitting is conducted using the candidate contour segments, the fitting degree between the candidate contour segments and fitted ellipse is not considered, which will result in a great deviation from the actual ellipse. In this work, to exclude the wrong candidate contour segments, the deviation error measurement is proposed to determine its fitness evaluation from the contour segments to the fitted ellipse. Thus, the deviation error measurement can be represented as

17 $$DEM(C{S}^{\#}{\boldsymbol{,}}CE)=E/{M}^{\#}=\lambda /{M}^{\#},$$

where *CS*^#^ denotes the data points of given contour segments, *CE* is the candidate ellipse, *E* is the sum of the least-squares algebraic distances of the data points to the ellipse and *M*^#^ is the total number of points on *CS*^#^.

If the value of the deviation error is less than the threshold, then the contour segment belongs to the contour of the same urediniospore; otherwise, it does not.

#### Steps of contour segment merging

Fig. 6d shows an example that illustrates the merging steps. As shown in Fig. 6d, the contour of the touching urediniospores was divided into eight contour segments. The merging steps of the contour segments of the touching urediniospores are as follows:

- Step 1: Select the longest segment of these contour segments *CS*_8_.
- Step 2: Set a distance measurement threshold *ω*_DM_ and measure the distance between the selected segment *CS*_8_ and each of the remaining segments *CS*_*i*_. Build a set CS^*^ if DM(*CS*_8_,*CS*_*i*_) < *ω*_DM_ is satisfied; that is,

18 $${C}{{S}}^{\ast }=\{{C}{{S}}_{i}|{\rm{DM}}({C}{{S}}_{8}{\boldsymbol{,}}{C}{{S}}_{i}) < {\omega }_{{\rm{DM}}},i=1,2,\ldots ,k\}.$$

Thus, a set CS^*^ is constructed with *CS*_4_, *CS*_5_, *CS*_6_ and *CS*_7_ because their distance measurements are smaller than *ω*_*DM*_.

- Step 3: Fit ellipses with *CS*_8_ and each segment in CS^*^ using the least-squares ellipse fitting algorithm, which is shown as the ellipses in Fig. 6e. Calculate the DEMs between these two contour segments (*CS*_8_ and *CS*_4_, *CS*_8_ and *CS*_5_, *CS*_8_ and *CS*_6_, and *CS*_8_ and *CS*_7_) and their corresponding fitted ellipses. Merge *CS*_8_ with all *CS*_*i*_ whose DEMs are smaller than *σ*_DEM_ to create *CS*_new_, which can be represented as

19 $${C}{{S}}_{{\rm{n}}{\rm{e}}{\rm{w}}}=\{{C}{{S}}_{8}+{C}{{S}}_{i}|{\rm{DEM}}({C}{{S}}_{8}{\boldsymbol{,}}{C}{{S}}_{i}) < {\sigma }_{{\rm{DEM}}}{\boldsymbol{,}}i=1{\boldsymbol{,}}2{\boldsymbol{,}}\ldots {\boldsymbol{,}}k\}.$$

Because there are no segments to satisfy the condition, *CS*_8_ is merged as a new contour segment.

- Step 4: Fit the ellipse with this new segment *CS*_new_ as the correct ellipse for one of the touching urediniospores shown as Fig. 6f, where the number of urediniospores is Num + 1, and delete *CS*_new_.
- Step 5: Delete *CS*_new_ from the contour segment list *CS*_*N*_. Check if all *CS*_*N*_ are deleted. If so, output all correct ellipses and end the program. If not, go back to Step 1, and the steps are iterated until all the contour segments are selected to fit the best representative ellipses (Fig. 6g).

**Figure 7:**
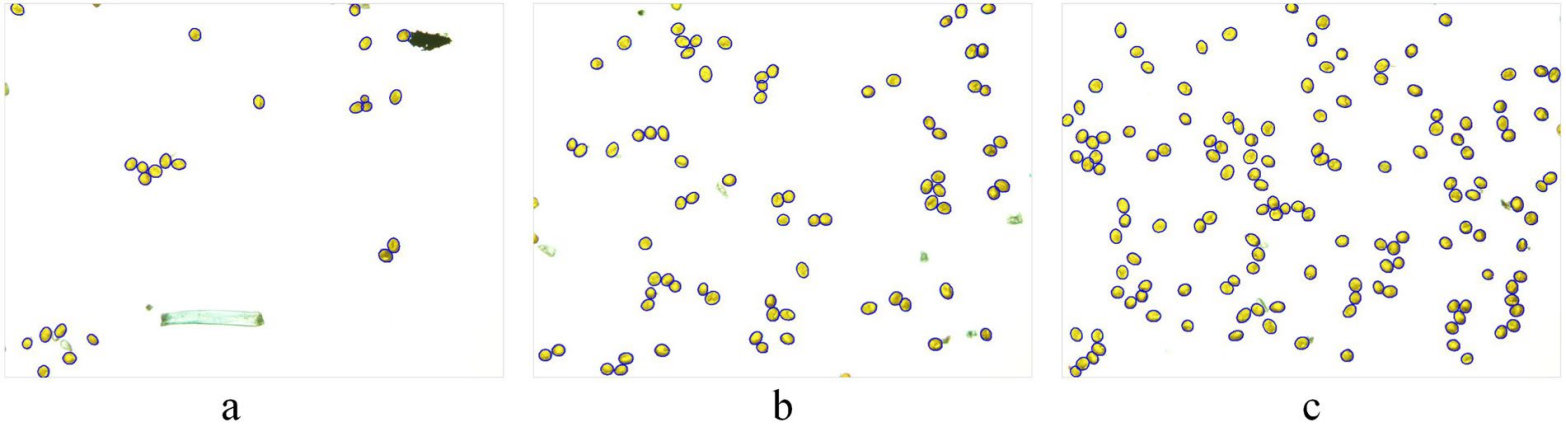
Segmentation effect and counting results of the original image of urediniospores under different capture times: (**a**) image after capturing for 5 min; (**b**) image after capturing for 10 min; (**c**) image after capturing for 15 min.

With 30 urediniospore image samples for statistical analyses, when the parameter *ω*_DM_ was less than 40, all contour segments of the same spore were included in CS^*^, and when the parameter *σ*_DEM_ was less than 95, the wrong candidate contour segments were excluded. Thus, *ω*_DM_ and *σ*_DEM_ were set to 40 and 95, respectively. The results of the proposed algorithm for segmenting and counting the image in Fig. 3 are shown in Fig. 7. This shows that the touching urediniospores were well divided.
